# The Anti-Inflammatory Role of GLP-1 RAs in Acute Lung Injury and Acute Respiratory Distress Syndrome

**DOI:** 10.3390/ijms27072922

**Published:** 2026-03-24

**Authors:** Paul Dumitrescu, Beata Kosmider

**Affiliations:** 1 Center for Inflammation and Lung Research, Lewis Katz School of Medicine, Temple University, Philadelphia, PA 19140, USA; 2 Department of Microbiology, Immunology, and Inflammation, Lewis Katz School of Medicine, Temple University, Philadelphia, PA 19140, USA

**Keywords:** ALI, ARDS, lung, GLP-1 RAs, mice, clinical trials, anti-inflammatory response, LPS, sepsis

## Abstract

Acute lung injury (ALI) and acute respiratory distress syndrome (ARDS) pose a significant burden on the healthcare system. The mechanisms underlying the pathophysiology of ALI/ARDS are widely studied. However, currently, there are no clinically approved drugs that can effectively reduce the high mortality of patients. Glucagon-like peptide-1 receptor agonists (GLP-1 RAs) are an increasingly popular class of medications. Their FDA approval was driven by the beneficial effects in patients with type 2 diabetes mellitus. Notably, recent studies are beginning to recognize the role of GLP-1 RAs in immunomodulation and anti-inflammatory responses across various organs, including the lungs. Animal models of ALI demonstrate the potential of these medications for treatment and prophylaxis. Observational studies suggest that patients taking GLP-1 RAs experienced fewer pulmonary complications. Here, we reviewed reports on their impact on the respiratory system in animal models of ALI and in clinical trials. Their effects in the intensive care unit setting and conditions predisposing to ALI/ARDS were also summarized. The mechanisms of action of GLP-1 RAs were reviewed based on in vitro studies using various lung cell types, and experimental approaches. Moreover, the roles of the pharmaceutical industry and patent law in extending the scope of GLP-1 RAs beyond obesity and diabetes were also described.

## 1. Introduction

Severe acute lung injury (ALI) can progress to acute respiratory distress syndrome (ARDS), a clinical syndrome of diffuse lung inflammation and edema that can result in respiratory failure [1]. It can be caused by a variety of factors, leading to direct lung injury via a local pro-inflammatory response or indirectly via systemic inflammatory and injury mediators. Pathogens can cause pulmonary sepsis, and among the non-infectious causes, pancreatitis, aspiration of gastric contents, and severe traumatic injuries with shock and multiple transfusions are the most common. Patients with direct pulmonary causes exhibit greater alveolar epithelial injury and inflammation than those with indirect non-pulmonary origins. Given that extrapulmonary causes of ARDS affect the whole lung via endothelial dysfunction rather than the more localized damage expected in a direct pulmonary cause, a more diffuse injury pattern is common. Both pulmonary and non-pulmonary sepsis can lead to ARDS, and alveolar fluid reduces the amount of oxygen in the bloodstream [1,2]. Studies conducted before the coronavirus disease 2019 (COVID-19) pandemic suggest that severe ARDS accounted for 23.4% of all intensive care unit (ICU) admissions [3]. Also, ARDS was the most common cause of ICU death, with a mortality rate ranging between 30 and 50% [4]. No approved pharmacologic therapy has been shown to consistently reduce mortality, while the treatment focuses on supporting the patient.

## 2. ALI/ARDS Pathophysiology

### 2.1. Historical and Clinical Perspective

ALI/ARDS is a class of pathophysiological syndrome that represents a spectrum of respiratory failure, bilateral pulmonary injury, and severe hypoxemia caused by non-cardiogenic pulmonary edema [5]. Clinical definitions of ARDS have had a unique history. Since the 20th century, worldwide pandemics have led to significant respiratory syndromes, and clinicians have described these phenomena in different ways. In the 1918–1919 influenza pandemic, ARDS was described as “purulent bronchitis”, “fulminating pneumonia”, and “lung full of hemorrhages.” These definitions were updated in the 1960s as “wet lung” and “shock lung.” Currently, diagnostic criteria rely on the American-European Consensus Conference, called the Berlin definition. There are four main clinical criteria defining ARDS:

- Timing: Within one week of a known clinical insult or new or worsening respiratory symptoms.
- Chest imaging: Bilateral opacities—not fully explained by effusions, lobar/lung collapse, or nodules.
- Origin of edema: Respiratory failure not fully explained by cardiac failure or fluid overload, with objective assessment to exclude hydrostatic edema if no risk factor is present.
- Oxygenation:
  - Mild: 200 mm Hg < PₐO_2_/FIO_2_ ≤ 300 mm Hg with PEEP or CPAP ≥ 5 cm H_2_O
  - Moderate: 100 mm Hg < PₐO_2_/FᵢO_2_ ≤ 200 mm Hg with PEEP ≥ 5 cm H_2_O
  - Severe: P_a_O_2_/FIO_2_ ≤ 100 mm Hg with PEEP ≥ 5 cm H_2_O

These clinical descriptions were agreed upon by a panel of experts convened in 2011 in Berlin, Germany [6]. It was an initiative of the European Society of Intensive Care Medicine with endorsement from the American Thoracic Society and the Society of Critical Care Medicine. The Berlin Definition draft was evaluated using patient-level meta-analysis of 4188 patients with ARDS from 4 multicenter clinical data sets and 269 patients with ARDS from 3 single-center data sets containing physiological information. Such understanding from a molecular biology perspective has been more elusive. Physiologically, ALI is studied in animals, where commonly an endotoxin, bacterial lipopolysaccharide (LPS), is used [7]. In these models, lung parenchyma is damaged by reactive oxygen and nitrogen species produced by activated lung macrophages and transmigrated neutrophils. This results in microvascular injury and diffuse alveolar damage with intrapulmonary hemorrhage, edema, and fibrin deposition.

### 2.2. Phase-Based Progression

The inflammatory pathways in ALI/ARDS can lead to widespread tissue damage and multisystem organ failure [8]. The pathology may progress through three overlapping phases: exudative, proliferative, and fibrotic. The exudative phase is the earliest and occurs within the first week of injury. It has been described as a disruption to the alveolar–capillary barrier that results in the accumulation of protein-rich fluid in distal airspaces and impaired gas exchange. The influx of neutrophils and the release of the pro-inflammatory factors exacerbate both epithelial and endothelial injury. From a physiologic perspective, features like hyaline membrane formation, pulmonary edema, and reduced lung compliance are hallmarks of this phase [9].

The proliferative phase follows the exudative phase and can take days to weeks [8,9,10]. Inflammatory cells continue to enter the alveolar spaces until neutrophils undergo apoptosis, and macrophages follow a transition from a pro-inflammatory M1 to an anti-inflammatory M2 phenotype. This pro-repair transition also supports the proliferation of alveolar epithelial type II (ATII) cells and a partial reconstruction of the alveolar barrier, which has been disturbed by fluid shifts during the exudative phase. The proliferative phase mediates resolution of the exudative phase effects, which may be delayed if oxidative stress and impaired phagocytic clearance persist [10]. The shift towards inflammation resolution may be orchestrated by specialized pro-resolving mediators (SPMs) such as resolvins and protectins. They are lipid-derived factors that function by actively inhibiting neutrophil infiltration, facilitating the clearance of apoptotic cells, and promoting the restoration of tissue homeostasis.

The fibrotic phase, which develops in only a subset of ARDS patients, is characterized by unrestrained fibroblast activity, excessive collagen deposition, and permanent structural changes in the lung [9]. This persistent fibroproliferation may lead to prolonged ventilation–perfusion mismatching and decreased lung compliance, resulting in severe respiratory dysfunction. This phase was of particular interest in the COVID-19 studies [1]. Increased fibrotic alterations were associated with disease severity, extended duration of ARDS, and refractory hypoxemia that was especially difficult to manage without risking ventilator-induced lung injury (VILI).

### 2.3. Alveolar–Capillary Barrier Dysfunction

The alveolar–capillary barrier regulates gas exchange and fluid balance [1]. It consists of layers of alveolar epithelial and capillary endothelial cells separated by a thin basement membrane. When the integrity of the alveolar–capillary barrier is compromised during ALI, it results in increased filtration of protein-rich edema into the interstitial and alveolar spaces [11]. It also reduces the alveolar epithelium’s ability to clear excess liquid. To further understand the shifts observed, particularly in the exudative and proliferative phases, it is necessary to examine cell phenotypes in ALI/ARDS.

The lung epithelium is composed of alveolar type I cells and ATII cells [1]. Damage to the epithelial barrier facilitates alveolar flooding and impairs fluid transport, the mechanism that maintains a dry airspace. ATII cell injury can affect surfactant production. Damage-associated molecular patterns (DAMPs) released into the airspace by epithelial cell necrosis and leakage of intracellular contents can amplify pro-inflammatory signaling. Studies investigating macrophage communication with other inflammatory cells have focused primarily on the acute phase, in which neutrophil recruitment is driven by IL-8 secretion [10]. Notably, the pathogenesis of ARDS is characterized by neutrophil-dependent lung injury [9]. Their accumulation leads to endothelial and alveolar epithelial damage, resulting in alveolar edema. Macrophages in ALI/ARDS display altered transcriptional and functional profiles that prolong inflammation [11]. Their polarization toward an M1 or M2 phenotype is likely induced during recruitment into the air spaces or within the inflamed alveolar compartment. It is associated with transcriptional programs induced by DAMPs and cell–cell interactions.

On the vascular side, the pulmonary endothelial cells adopt a pro-inflammatory phenotype, characterized by increased permeability and upregulation of intercellular adhesion molecule 1 (ICAM-1) and vascular cell adhesion molecule 1 (VCAM-1) [12]. Loss of intercellular tight junctions, which include zonula occludens (ZO-1) and occludin, as well as adherens junctions containing vascular endothelial (VE)-cadherin, contributes to the pulmonary microvascular leakage in ALI. The pro-inflammatory phenotype of endothelial cells can initiate lung parenchymal inflammation once activated by harmful stimuli. They can produce chemokines (e.g., CXCL1, CXCL2, and CXCL8), which in turn promote inflammatory cell infiltration and alveolar–capillary barrier damage in ALI.

In summary, structural and functional disruption of alveolar epithelial cells compromises the integrity of the alveolar–capillary barrier, leading to pulmonary edema, impaired gas exchange, and the amplification of inflammatory cascades [13]. They exhibit oxidative damage and mitochondrial dysfunction, which can lead to various forms of cell death, impaired fluid clearance, and regenerative capacity. This pathophysiological cascade is driven by multiple mechanisms reviewed below.

### 2.4. The Pro-Inflammatory Response

ALI/ARDS is driven by intense inflammation involving both innate and adaptive immune responses [14]. The inflammatory cascade begins with recognition of DAMPs and pathogen-associated molecular patterns (PAMPs) by pattern recognition receptors (PRPs), including Toll-like receptor 4 (TLR4) on alveolar macrophages and epithelial cells. This activates intracellular signaling via nuclear factor-κB (NF-κB) and mitogen-activated protein kinase (MAPK), leading to the transcription of pro-inflammatory mediators such as tumor necrosis factor-α (TNF-α), interleukin (IL)-1β, and IL-6. They promote early-phase vascular leakage, immune cell recruitment, and tissue damage—all hallmarks of ALI/ARDS. Studies indicated that suppression of NF-κB pathways in macrophages in LPS-induced ALI in mice and rats alleviated inflammation [15,16]. NF-κB activity was increased in human macrophages exposed to LPS [17]. This provides new insights into disease pathophysiology.

Moreover, IL-6, IL-8, IL-10, and TNF-α secreted by alveolar macrophages in the airspace stimulate chemotaxis and activate neutrophils [18]. One neutrophil mediator, elastase, may degrade epithelial junctional proteins, possess pro-apoptotic properties, and exert cytotoxic effects on the epithelium. Alveolar epithelial cell injury disrupts normal fluid transport by downregulating epithelial Na^+^ channels and Na^+^/K^+^-ATPase pumps, impairing the resolution of alveolar flooding.

In a mouse model of ALI induced by acid aspiration, alveolar macrophage-derived TNF p55 receptor promoted pulmonary edema formation, specifically through activation of death signaling [19]. The resultant caspase-8 activation in alveolar type I cells contributed to lung injury and dysfunction. These findings defined the pathophysiology and physiological alterations in early ALI.

Pro-inflammatory signals dominate the early phase of ARDS. However, if the proliferative phase is extended or disrupted, growth factors can become excessive, leading to lung fibrosis [9]. The increase in post-ARDS fibrosis seen during the COVID-19 pandemic, even with lung-protective ventilation techniques, highlights the need for further research into its spectrum and phenotyping.

A subset of ARDS patients has been shown to exhibit a distinct hyperinflammatory subphenotype [1]. It is characterized by higher plasma expression of IL-6, IL-8, and tumor necrosis factor receptor 1 (TNFR1), and lower levels of bicarbonate and protein C compared with the hypoinflammatory subphenotype. Patients with the hyperinflammatory subphenotype more often have extrapulmonary sepsis as a risk factor for ARDS. However, characterizing ALI/ARDS patients based on biological phenotypes is limited because lung biopsies are only performed in selected cases of non-resolving ARDS, and autopsy findings represent the most severely ill patients who did not survive.

## 3. The Anti-Inflammatory Role of GLP-1 RA

### 3.1. Historical Perspective

Endogenous glucagon-like peptide-1 (GLP-1) was discovered in the 1980s and observed to have an incretin-like effect [20]. It is secreted by intestinal L-cells and by neurons in the brain, including the hypothalamus. GLP-1 increases in plasma within minutes of eating a carbohydrate- or fat-containing meal [21]. Physiologically, it stimulates insulin synthesis and suppresses glucagon secretion. GLP-1 has an appetite suppressant effect through both the peripheral and central nervous system pathways [20]. One part of this mechanism is the slowing of gastric emptying, stimulation of vagal afferent signals to the solitary nucleus of the medulla, and their projection to the appetite centers of the hypothalamus to induce satiety, or to the area postrema to cause nausea.

There were limited pharmacological uses of GLP-1 due to its rapid degradation by dipeptidyl peptidase-4 (DPP-4) and its short half-life of 2 min [20,22]. However, the potential for GLP-1 receptor pharmacological manipulation was evident, with the discovery of exendin-4 from the venom of the Gila monster, *Heloderma suspectum.* Exendin-4 stimulated the GLP-1 receptor and was resistant to DPP-4 degradation while having a much longer half-life. Exenatide, a synthetic peptide identical to exendin-4, was approved for the treatment of type 2 diabetes mellitus (T2DM) as monotherapy and later as an add-on treatment. Liraglutide is a long-acting GLP-1 receptor agonist (GLP-1 RA). A GLP-1 peptide sequence was modified at the N-terminus to resist degradation by DPP-4. Fatty acids were added to enhance protein binding and prolong half-life. GLP-1 RAs are an increasingly diverse class of medications with different mechanisms, routes of delivery, and side effects [23]. FDA-approved GLP-1 RAs for glycemic control or weight loss include: dulaglutide, exenatide, liraglutide, liraglutide/insulin degludec, lixisenatide/insulin glargine, semaglutide, and tirzepatide (dual glucose-dependent insulinotropic peptide—GIP and GLP-1 RA).

Though the beneficial effects in T2DM patients drove FDA approval, recent studies are beginning to recognize the role of GLP-1 and GLP-1 RAs in the setting of immunomodulation and anti-inflammatory responses [24]. Interest in GLP-1 RAs has also emerged in ICU settings, where they have been shown to exert broad beneficial effects across multiple organ systems through various mechanisms [25].

### 3.2. Methods

The studies in the PubMed database were systematically searched. Reports on the role of GLP-1 RAs were included. Specifically, we reviewed recent studies focused on their effects in ALI/ARDS and predisposing conditions in the respiratory field. Initial screening of titles and abstracts was conducted to identify relevant articles. Searches were also performed by analyzing references in publications. Studies on GLP-1 RAs mechanisms of action, both in vitro and in vivo, meta-analyses, and clinical trials were reviewed. In addition, we included reports from human subject studies that used GLP-1 RAs for which respiratory condition information was available.

### 3.3. The Effect of GLP-1 RAs in Clinical Trials

Although specific attention was paid to GLP-1 RAs and to patients with comorbid T2DM and obesity, their potential therapeutic role in lung diseases was also suggested [26]. The lung has been identified as an organ of high GLP1R mRNA (messenger RNA) expression. The presence of the peptide itself may be a biomarker of worsening severity in acute respiratory failure [27]. It was also found that elevated GLP-1 levels were associated with a greater need for mechanical ventilation. Notably, this correlation appears specific to GLP-1 rather than to other incretins, such as GIP, reinforcing GLP-1’s role in the pathophysiology of critical illness. While seemingly counterintuitive, elevated GLP-1 may represent a compensatory anti-inflammatory response in these pro-inflammatory conditions, thereby allowing GLP-1 RAs to exploit this pathway pharmacologically to reduce inflammation further [28]. This is supported by findings that they suppress cytokines, such as TNF-α, IL-6, and IL-1β, by inhibiting the NF-κB pathway [29].

Exenatide reduced HbA1C, systolic blood pressure, triglycerides, and high-sensitivity C-reactive protein (CRP) in obese patients with T2DM [30]. Also, it had an anti-inflammatory effect at the cellular and molecular levels, which may be beneficial for various organs [31]. There was a significant decrease in ROS levels, NF-κB, TNF-α, IL-1β, c-Jun N-terminal kinase (JNK), TLR-2, TLR-4, and suppressor of cytokine signaling 3 (SOCS-3) in mononuclear cells after 12 weeks of treatment. It was also reported that GLP-1 RAs enhanced mitochondrial function and reduced oxidative stress in human peripheral blood polymorphonuclear leukocytes [32]. This reduced leukocyte–endothelial interactions and inflammation in T2DM.

Small-scale randomized controlled studies in humans examined the impacts of GLP-1 RAs on lung mechanics, with significant findings in increased surfactant production by ATII cells and improvements in forced vital capacity (FVC) [33]. This highlights the potential of GLP-1 RAs to reduce injury to the alveolar–capillary barrier, a critical feature of ALI pathophysiology.

Seven trials, involving 55,922 participants, were included in the meta-analysis [34]. There were 27,942 subjects in the GLP-1 RA group and 27,980 in the placebo group. This extensive study was designed to assess the association between GLP-1 RAs use and 12 types of respiratory disorders. Although not statistically significant, GLP-1 RAs versus placebo showed a reduced trend in the risk of 9 respiratory disorders (Table 1). Notably, no or only mild heterogeneity was observed in any of the meta-analyses, and all the original studies included were of high quality. However, the incidence rate of all respiratory disorders of interest was low. Future studies with large sample sizes and long-term follow-up are needed for higher event rates and to validate the findings. Another meta-analysis of 28 randomized controlled trials involving 77,485 participants with T2DM investigated the role of GLP-1 RAs (Table 1) [35]. A reduction in the risk of respiratory disease (RR 0.86) was found, especially for pulmonary edema and bronchitis.

A high death rate in COVID-19 patients with multiple comorbid conditions was observed [42,43]. However, hospitalized diabetic patients with COVID-19 on GLP-1 RAs before admission had a reduced mortality rate, with an odds ratio (OR) of 0.53, compared with those on non-GLP-1 RA diabetic medications. This effect persisted even after adjustment for potential confounding factors. The anti-inflammatory effect of GLP-1-RAs could reduce the exaggerated inflammatory response caused by SARS-CoV-2 infection. It was indicated that patients on GLP-1 RAs may have survival benefits from NF-κB inhibition, which is part of the NLR family pyrin domain-containing 3 (NLRP3) inflammasome. The *NLRP3* gene is a direct target of NF-κB due to binding sites in its promoter region [42]. The inflammasome receptors oligomerize and recruit procaspase-1, triggering its conversion to active caspase-1, which cleaves pro-IL-1β and pro-IL-18 into their mature forms. Therefore, it was suggested that, because GLP-1 RAs interfere with the NF-κB signaling, they could also reduce NLRP3-mediated inflammation and the pro-inflammatory cytokine storms observed in advanced stages of COVID-19. Moreover, the pleiotropic activity of GLP-1 RAs may be further beneficial in the management of SARS-CoV-2 infection. The protective role of angiotensin-converting enzyme 2 (ACE2) in ARDS, partly due to restored angiotensin-1–7 production, has been reported [44]. During COVID-19, GLP-1 RAs have been shown to potentially increase ACE2 expression [42]. This could ameliorate lung injury by antagonizing the reduction in ACE2 levels, a hallmark of SARS-CoV-2 infection progression, and by precluding the immune response critical for ARDS. On the other hand, ACE2 enables viral entry into host target cells, raising concerns about increased vulnerability to infection in the case of its upregulation. From a mechanistic standpoint, further studies are needed to define the role of GLP-1 RAs in SARS-CoV-2 infection.

Adrenal glucocorticoid production is induced upon activation of the hypothalamic–pituitary–adrenal (HPA) axis, which coordinates physiological responses to external stimuli [45]. The effects of prolonged exposure to GLP-1 RA on cortisol metabolism in humans were analyzed [46]. HPA axis activity was studied after 3 weeks of treatment with the 1.5 mg dulaglutide compared with placebo in healthy volunteers. Urinary free cortisol, the circadian rhythm of serum and salivary cortisol, cortisol after a 1 mg dexamethasone suppression test, and cortisol levels before and after adrenocorticotropic hormone (ACTH) stimulation were analyzed. This drug did not affect the HPA axis like that observed during a chronic stress-like state. These results indicate its beneficial properties for long-term treatment.

Together, clinical observations of GLP-1 RAs showed some amelioration of COVID-19 pathology and other benefits in ALI/ARDS. Studies indicate that GLP-1 RAs have pleiotropic effects and affect multiple pathways. Additionally, injury to various cell types is implicated in ARDS, making GLP-1 RAs potentially beneficial candidates for its treatment. The severity of ARDS and its high mortality, coupled with the lack of effective pharmacological interventions, warrant further investigation into the mechanisms by which GLP-1 RAs act in the pulmonary setting.

### 3.4. Clinical Considerations in the ICU

There are diverse clinical, physiological, and molecular characteristics of ARDS [9]. Although advances in management and logistics of intensive care (e.g., prone positioning) have offered some opportunities for improvement, pharmacological interventions have been largely unsuccessful. GLP-1 RAs have promise as multiorgan protective agents in critical illness, offering benefits beyond glucose control [24]. Their anti-inflammatory and metabolic protective properties may prevent or attenuate organ failure, support recovery, and improve long-term outcomes in critically ill patients. The study was conducted to determine whether the use of GLP-1 RAs before ICU admission might be associated with a higher incidence of clinically significant gastrointestinal outcomes [25]. The relationship between GLP-1 RAs use and the incidence of nausea/vomiting, constipation, ileus, obstruction, impaction, aspiration pneumonia, or gastroparesis, as well as hospital and ICU length of stay and mortality, was determined. This retrospective study included patients admitted to the ICU who had obesity or diabetes mellitus. Of the 31,327 eligible patients, 631 had been exposed to GLP-1 RAs before ICU admission. It was determined that GLP-1 RA exposure was not associated with gastrointestinal complications, ICU/hospital mortality, or ICU/hospital-free days in subgroups of patients who underwent surgery within 48h of admission and those who did not. Most GLP-1 RAs scripts were for dulaglutide (N = 244), followed by semaglutide (N = 241). The only statistically significant finding was a decrease in ICU mortality among those exposed to dulaglutide. Finally, initiation of a GLP-1 RAs within 30 days of ICU admission was associated with a significantly greater number of ICU-free days. These results highlight their potential role in improving hospital outcomes.

Furthermore, GLP-1 RAs can have significant implications for the management of patients under anesthesia, particularly regarding gastric emptying, enteral nutrition tolerance, and glycemic control [47]. The cross-sectional study investigated the association between the use of these drugs and residual gastric content (RGC). In fasted patients presenting for elective procedures under anesthesia, once-weekly GLP-1 RA use was associated with a significantly higher prevalence of increased RGC on preprocedural gastric ultrasonography (GUS) [48]. However, whether this has any impact on aspiration during general anesthesia is unknown [49]. Future studies are needed to evaluate safe discontinuation intervals and preprocedural fasting times for patients on GLP-RAs before elective procedures under anesthesia, efficacy, and optimal application across ICU populations. Although results from observational clinical studies are valuable, randomized trials specifically designed for ALI/ARDS would be useful for comparing the effects of GLP-1 RAs and for confirming causality. Studies with large sample sizes and long-term follow-up are also needed.

### 3.5. The Role of GLP-1 RAs In Vitro and In Vivo

GLP-1 RAs exhibit significant immunomodulatory properties across a range of cell types that could be relevant to the pathogenesis of ALI/ARDS [50,51,52]. The mechanism by which exendin-4 inhibited LPS-induced iNOS expression was investigated in murine RAW 264.7 macrophages [53]. Its protein levels were reduced, and the drug’s effect was mainly dependent on cAMP/PKA. It also decreased LPS-induced oxidative stress, TNF-α, IL-1β, IL-6, matrix metalloproteinase 2 (MMP-2), and MMP-9 expression, and JNK and AP-1 pathway activation [54]. Exenatide significantly increased IL-10 levels and decreased TNF-α and IL-1β expression in LPS-treated human monocytes/macrophages [55]. It skewed macrophage phenotype toward an anti-inflammatory state, with this effect predominantly attributable to PKA. T lymphocytes modulate the inflammatory response, and exendin-4 promoted T regulatory cells (Tregs) [56].

Studies using mouse models have shown that GLP-1 RAs can attenuate sepsis-induced ALI (Table 1) [4]. A two-hit model of sepsis and hyperoxia was used, and mice were pretreated twice daily with subcutaneous liraglutide injections. Compared to saline treatment, this drug improved sepsis-related pathophysiology and decreased alveolar–capillary barrier disruption, lung inflammation, and injury. These beneficial effects were independent of weight loss. Pretreatment with liraglutide was used in a study of LPS-induced ALI in mice (Table 1) [36]. Reduced the lung damage score, wet/dry weight ratio, IL-1β, IL-18 levels, and the total cell and protein content in bronchoalveolar lavage (BAL) fluid were detected compared to the LPS group. Also, decreased expression of NLRP3 and ASC proteins was observed in lung tissue. Myeloperoxidase (MPO) activity was lowered by liraglutide, suggesting that this may be a pathway through which the drug attenuated ALI. Also, liraglutide prevented LPS-induced polymorphonuclear neutrophil extravasation, lung injury, and alveolar–capillary barrier dysfunction (Table 1) [12]. The results showed that pulmonary inflammation, edema, increased insulin levels, alveolar cell injury, and reduced SP-A expression were markedly attenuated by this drug (Table 1) [37]. This was mediated via the thyroid transcription factor (TTF-1) signaling pathway. It has previously been shown that the TTF-1 response element is critical for regulating *SP-A2* promoter activity [57]. In another study of LPS-induced ALI in mice, liraglutide significantly lowered the lung injury score, wet/dry lung weight ratio, immune cell counts in BAL fluid, protein concentration, and cell apoptosis, and was associated with decreased gene expression of inflammatory cytokines and chemokines (Table 1) [38]. Notably, these effects were absent in GLP-1R KO mice. GLP-1 and GLP-based drugs in pancreatic β-cells reduce high-glucose-stimulated expression of thioredoxin-interacting protein (TxNIP), a key component of the inflammasome. LPS-challenged lungs showed elevated TxNIP mRNA and protein levels, which were diminished by this drug in a GLP-1R-dependent manner. This indicates that GLP-1R is crucial for mediating the drug’s beneficial effects in ALI, with TxNIP as a potential target.

Liraglutide also attenuated *Pseudomonas aeruginosa*-induced ALI in mice (Table 1) [39]. It prolonged survival, reduced TNF-α, IL-2, and IL-6 production, cell apoptosis, and edema. The mechanism was further explored in MLE-12 cells treated with this drug after LPS stimulation. Autophagy inhibition and restored pulmonary surfactant secretion were observed. In another study of mice infected with *Pseudomonas aeruginosa,* liraglutide reduced the levels of inflammatory cells, TNF-α, and IL-6 in the lungs compared with septic controls (Table 1) [40]. Notably, higher expression of SP-A and SP-B was detected, indicating a beneficial effect of the drug on the lung. Murine primary ATII cells pretreated with liraglutide also showed improved SP-A and SP-B expression after LPS treatment.

A reduction in the number of *Alternaria* extract-induced total BAL cells, including macrophages, eosinophils, and lymphocytes, was observed after mice treatment with liraglutide [52]. It suppressed acute eosinophilic lung inflammation and airway mucus production in innate allergic immune responses. This suggests that GLP-1 RAs play a beneficial role in reducing the pro-inflammatory response.

The impact of dulaglutide was analyzed in LPS-induced ALI in mice (Table 1) [41]. This drug reduced lung injury and cell apoptosis, alleviated pulmonary inflammation, and reversed increases in IL-1β, TNF-α, IL-6, CXCL1, CCL2, and CXCL2 expression, thereby reducing neutrophil and macrophage infiltration. This was caused by inhibition of signal transducer and activator of transcription 3 (STAT3), a crucial transcriptional regulator of the inflammatory response. Also, dulaglutide inhibited STAT3 phosphorylation, thereby preventing its nuclear entry and activation of target genes.

In the normal wound healing process, myofibroblasts are eliminated by apoptosis; however, in the fibrotic state, they become resistant and persist over time, promoting excessive extracellular matrix (ECM) deposition [58]. Studies in bleomycin-induced pulmonary fibrosis in rats showed that liraglutide decreased mRNA expression of collagen, hydroxyproline, and key collagen-synthesizing enzymes. Additionally, GLP-1R activation restored *ACE2* mRNA levels, modulated the activities of components of the renin–angiotensin system (RAS), increased surfactant protein expression, and improved pulmonary function, including a partial restoration of lung alveolar structure. Also, adipose tissue macrophages are associated with insulin resistance and fibrosis in humans [59]. The impact of dulaglutide was studied in a high-fat diet-induced lung fibrosis in mice, and a significant decrease in the fibrosis area was observed after this treatment [60].

**Figure 1:**
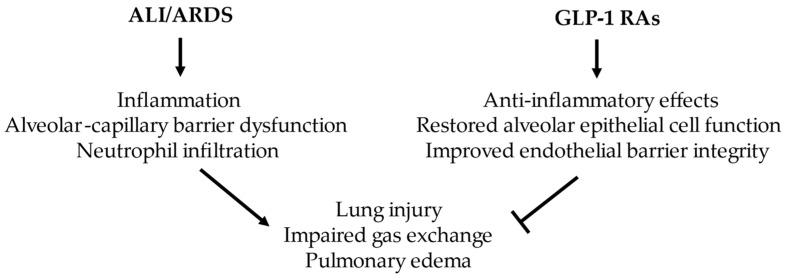
The beneficial role of GLP-1 RAs in ALI/ARDS models.

These findings indicate the beneficial effects of GLP-1 RAs and offer promise in treating metabolic disorders and various inflammatory diseases, such as sepsis, organ injury, and dysfunction [28,32,50,61]. They appear to coordinate anti-inflammatory responses across multiple cell types in ALI/ARDS and increase preservation of the epithelial and endothelial barriers (Figure 1). Notably, GLP-1 RAs’ potential lies in delaying a pro-fibrotic, accumulated state. As such, they may modulate the pulmonary microenvironment to favor resolution after injury.

## 4. Pharmaceutical Industry

Estimating the total costs of ARDS care has been difficult due to many factors, including the heterogeneity of provider systems, healthcare payment methods, and insurance policies [62]. Inpatient costs may be higher than those for other ICU syndromes. This is especially important as patients with ARDS related to COVID-19 have overwhelmed health systems in many parts of the world. Understanding both the health care and societal costs associated with ARDS is crucial for developing cost-effective interventions.

Patents are government-granted rights that typically last 20 years, allowing pharmaceutical manufacturers to exclude potential competitors from producing an entity that often includes not only the drug’s active ingredient but also its specific formulation, method of use, and delivery devices [63]. In a study examining 10 of the most popular FDA-approved GLP-1 RA drugs, the average medication had 19.5 patents, with the majority (54%) being on the delivery device rather than the active ingredient. The median patent protection time for GLP-1 RAs was 18.3 years, a significant factor in the cost of these medications. Further studies are needed to determine their effects alone and in combination as anti-inflammatory drugs in ALI/ARDS. Randomized controlled trials may further define their clinical impact.

In summary, GLP-1 RAs are an increasingly popular class of medications that are becoming widely available. They have offered incredible promise in the treatment of obesity and T2DM, and their anti-inflammatory and organ-protective properties are being studied in a variety of settings [24]. GLP-1 RAs show advantages in the management of ALI/ARDS, and given the high mortality rate, their potential benefits are critical.

## 5. Future Directions

There is great interest in the anti-inflammatory effects of GLP-1 RAs. However, they are an increasingly diverse class of medications with different mechanisms, routes of delivery, and side effects [23]. Most of them are administered through frequent subcutaneous injections [64]. The feasibility of GLP-1 RAs inhalations using various formulations and platforms was investigated [21,64]. Sustained-release GLP-1 RA-loaded microspheres have been explored. Improving drug bioavailability through the functional design of novel delivery systems, improving patient compliance, and translating these advances to the clinic are among the future directions. Numerous studies and clinical trials have evaluated GLP-1 RAs for their efficacy, safety, and tolerability. Still, additional evaluation, especially in the pulmonary setting, is needed to define their mechanisms of action and effects. Furthermore, the economic ramifications of healthcare should be considered. The high cost of pharmacological innovation and GLP-1 RAs create burdens on health insurance, taxpayer-funded health programs, and the economic well-being of patients [65]. Historically limited pharmacological options, along with the scale of clinical necessity, merit drug repurposing.

## Figures and Tables

**Table 1:** GLP-1 effects in ALI/ARDS and related conditions.

Treatment	System	Effect	Route	References
GLP-1 RAs	A meta-analysis of large, randomized trials	Reduced risk of pulmonary edema, pneumonia, acute respiratory failure, bronchitis, upper respiratory tract infection, squamous cell carcinoma of the lung, asthma, COPD, and pulmonary fibrosis.	Oral, subcutaneously.	[34]
GLP-1 RAs	Randomized controlled trials were analyzed	Lower risk of overall respiratory diseases, especially pulmonary edema.	Oral, subcutaneously.	[35]
Liraglutide	ALI in a two-hit model (sepsis and hyperoxia) in C57BL/6 mice	Reduced lung inflammation, alveolar–capillary barrier disruption, and lung injury.	Subcutaneously.	[4]
Liraglutide	LPS-induced ALI in BALB/c mice	Inhibition of the MPO activity, IL-18, and NLRP3 inflammasome pathway.	Subcutaneously.	[36]
Liraglutide	LPS-induced ALI in mice	Prevented polymorphonuclear neutrophil–endothelial adhesion by inhibiting the expression of ICAM-1 and VCAM-1.	Subcutaneously.	[12]
Liraglutide	LPS-induced ALI in BALB/c mice	Enhanced SP-A expression in ATII cells and attenuated pulmonary inflammation through the TTF-1 signaling.	Intraperitoneal injection.	[37]
Liraglutide	LPS-induced ALI in C57BL/6 mice	Reduced lung injury, wet/dry weight ratio, BAL fluid immune cell count, protein concentration, apoptosis, inflammatory cytokine and chemokine gene expression.	Subcutaneously.	[38]
Liraglutide	*Pseudomonas aeruginosa*-induced ALI in C57BL/6 mice	Prolonged survival, reduced the wet/dry lung weight ratio, pro-inflammatory responses, and attenuated pulmonary edema.	Subcutaneously.	[39]
Liraglutide	ALI model of pneumonia-induced sepsis in FVB/NJ mice	Decreased levels of inflammatory cells, TNF-α, and IL-6, and increased SP-A and SP-B levels and phospholipid secretion.	Subcutaneously.	[40]
Dulaglutide	LPS-induced ALI in C57BL/6 mice	Decreased IL-1β, TNF-α, IL-6, CXCL1, CCL2, CXCL2 levels, infiltration of neutrophils and macrophages, apoptosis, and STAT3 signaling.	Intraperitoneal injection.	[41]

## Data Availability

No new data were created or analyzed in this study. Data sharing is not applicable to this article.
